# Supplementary figures and images for: A novel behavioral paradigm using mice to study predictive postural control
Source: Front Neurosci. 2026 Apr 13;20:1790603. doi: 10.3389/fnins.2026.1790603 (PMC13111203; doi:10.3389/fnins.2026.1790603)

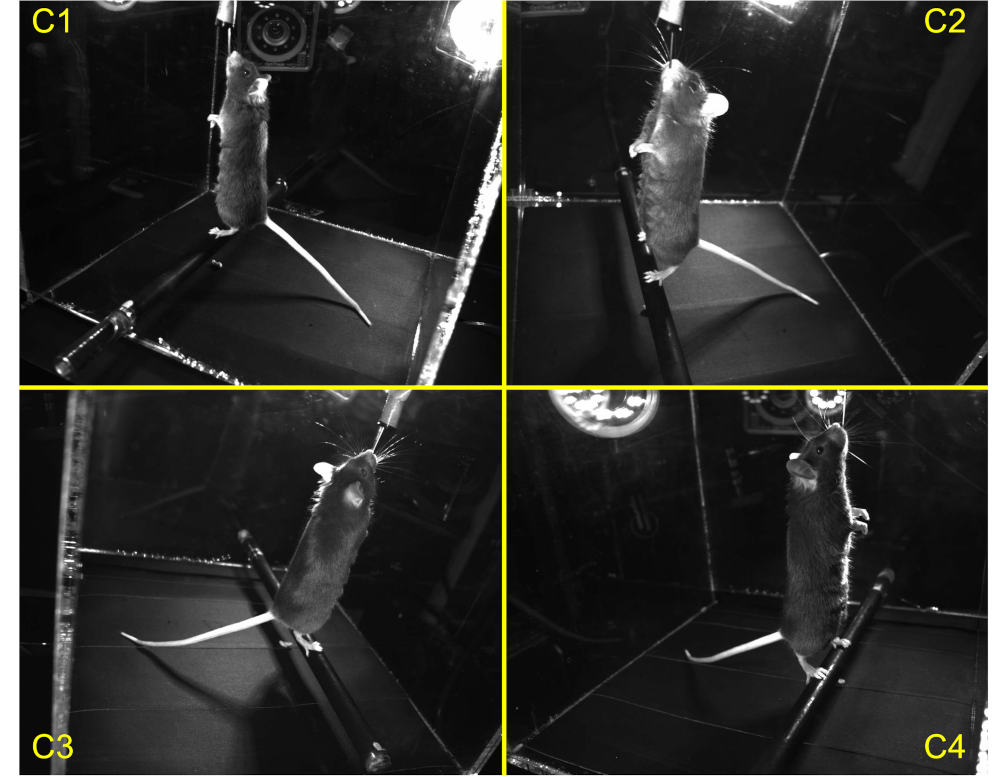

Supplement: SUPPLEMENTARY FIGURE S1 — Views from four cameras. An example simultaneous view from four cameras during the task. The analysis in this paper uses C4 camera only. [file Image_1.tif]

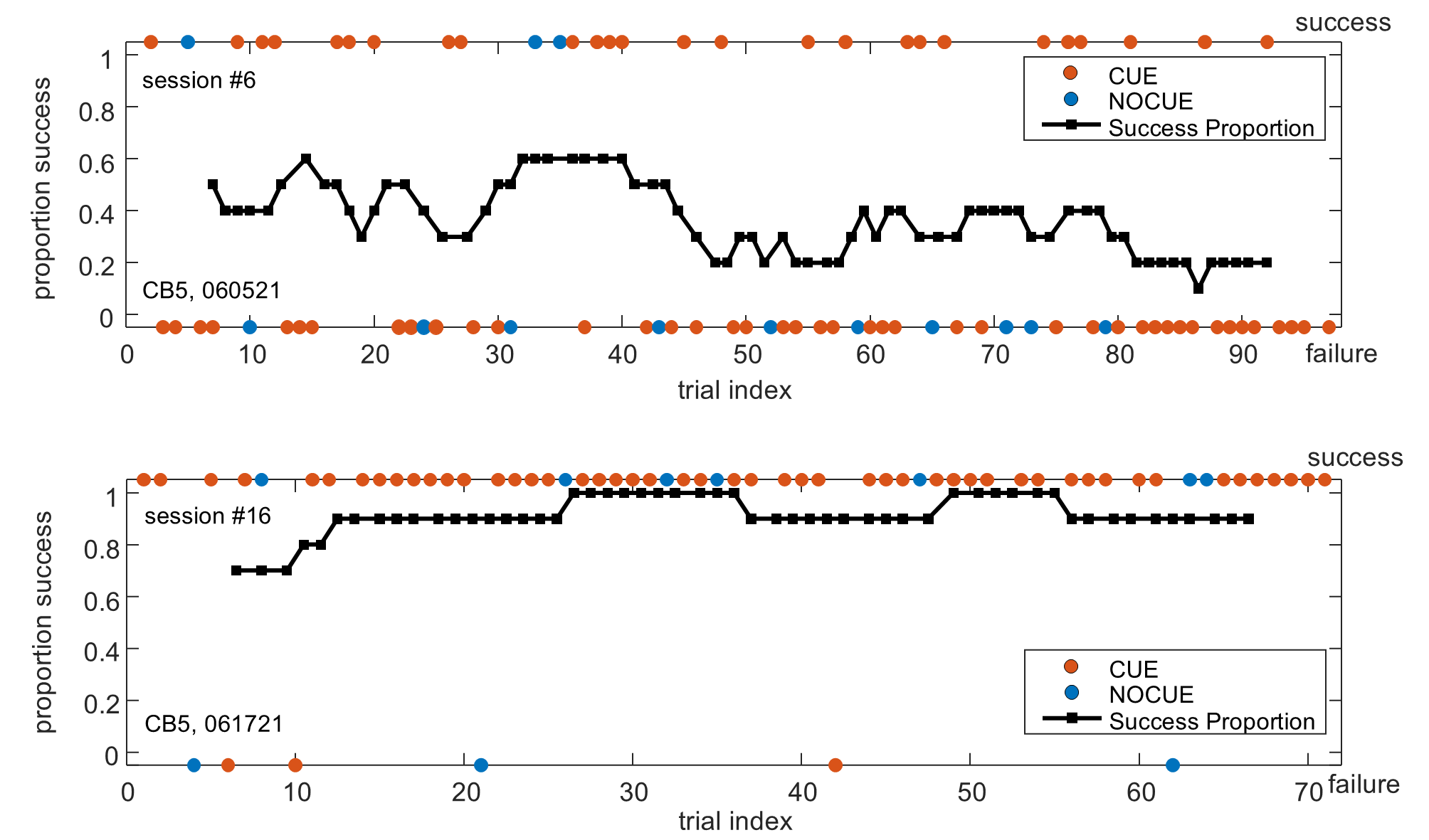

Supplement: SUPPLEMENTARY FIGURE S2 — Data analysis based on success proportion. This figure shows data from an example mouse (mouse CB5). The sliding window proportion of successes (sliding window size = 10, black) and every trial outcome (colored circles) in the first (top) and last (bottom) session of the large perturbation sessions. [file Image_2.tif]

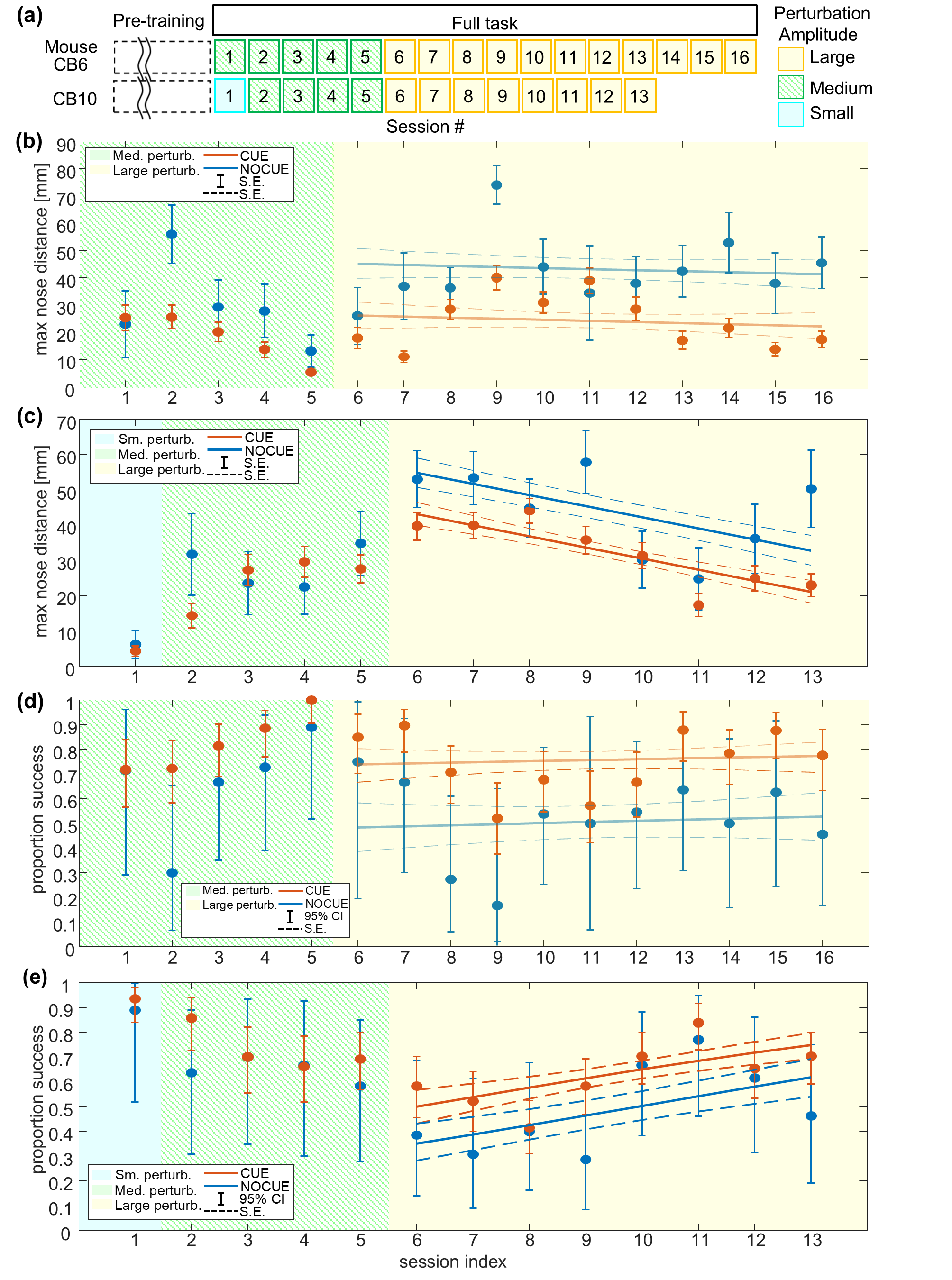

Supplement: SUPPLEMENTARY FIGURE S3 — Data analysis based on nose distance and success proportion for two additional mice. This figure shows data from two additional mice (mouse CB6 and CB10). (a) Time course of sessions. The platform was moved in one of three amplitudes: 7 (small), 12 (medium), or 18 (large) mm. Only one amplitude was used for an individual session. (b) Mouse CB6. Mean and standard errors of the max nose distance across all the sessions. Solid lines show fit from a linear mixed-effects model. Dashed lines represent standard errors for fits. Lines are translucent to indicate that the p-value for the session index coefficient was >0.05. (c) Mouse CB10. Mean and standard errors of the max nose distance across all the sessions. Solid lines show fit from a linear mixed-effects model. Dashed lines represent standard errors for fits. (d) Mouse CB6. The success proportion with 95% confidence intervals across all sessions. Solid lines show fits from a logistic mixed-effects model for CUE and NOCUE conditions. Dashed lines represent standard errors for fits. Lines are translucent to indicate that the p-value for the session index coefficient was >.05. (e) Mouse CB10. The success proportion with 95% confidence intervals across all sessions. Solid lines show fits from a logistic mixed-effects model for CUE and NOCUE conditions. Dashed lines represent standard errors for fits. [file Image_3.tif]

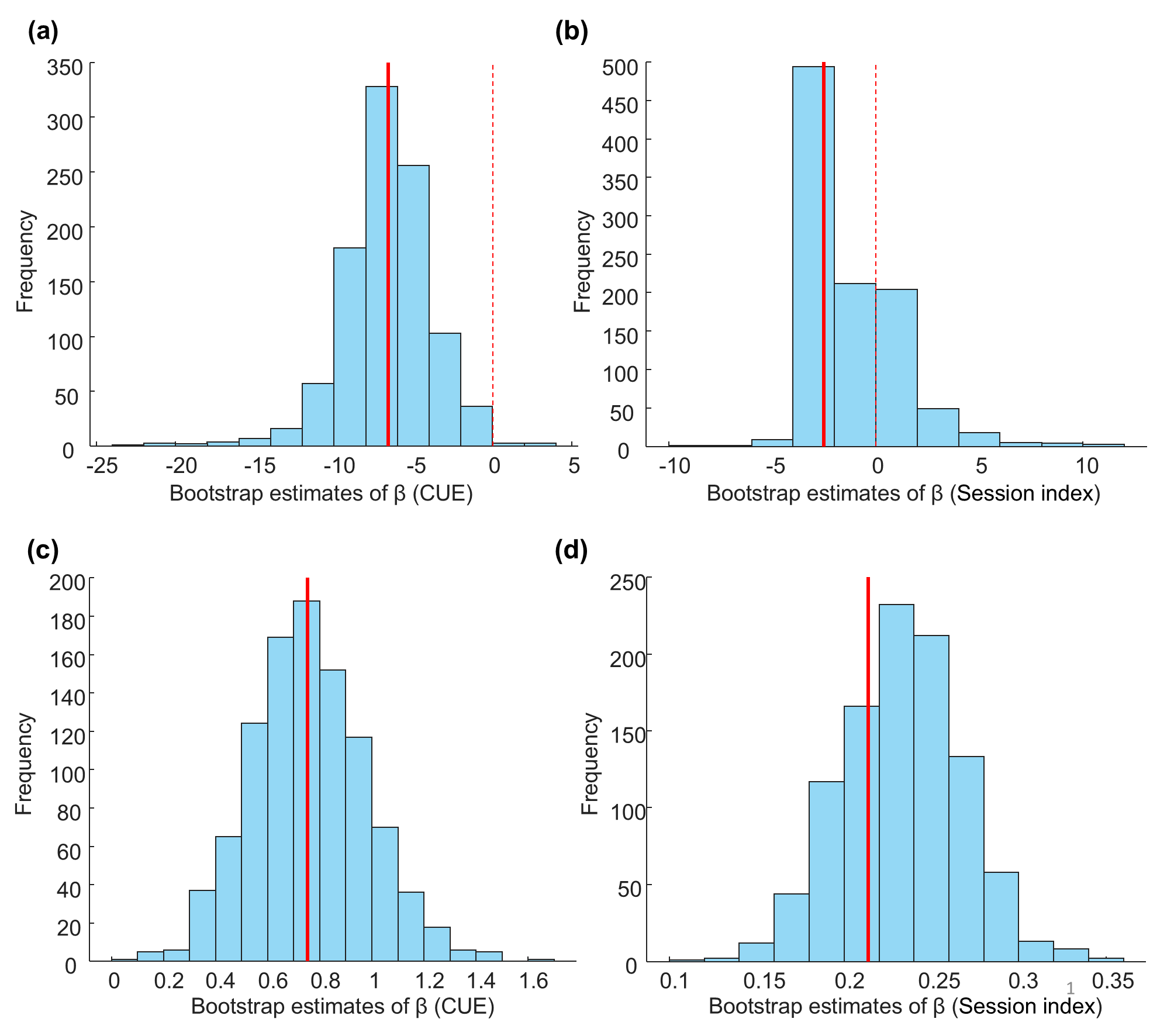

Supplement: SUPPLEMENTARY FIGURE S4 — Bootstrap distributions of fixed-effect estimates from mixed-effects models. Bootstrap distributions of the fixed-effect coefficients for CUE and session index predictors (mouse CB5). To assess robustness to imbalance in trial types, we repeatedly refit the model using balanced resampled subsets (n = 1000 iterations). Histograms show the distribution of bootstrap estimates of the fixed-effect coefficient (β). Solid red vertical lines indicate the coefficient estimated from the full dataset. (a,b) Distributions of coefficient estimates obtained from linear mixed-effects models. Dashed red lines indicate the null value (β = 0). (c,d) Distributions of coefficient estimates obtained from logistic mixed-effects models (binomial distribution, logit link). [file Image_4.tif]

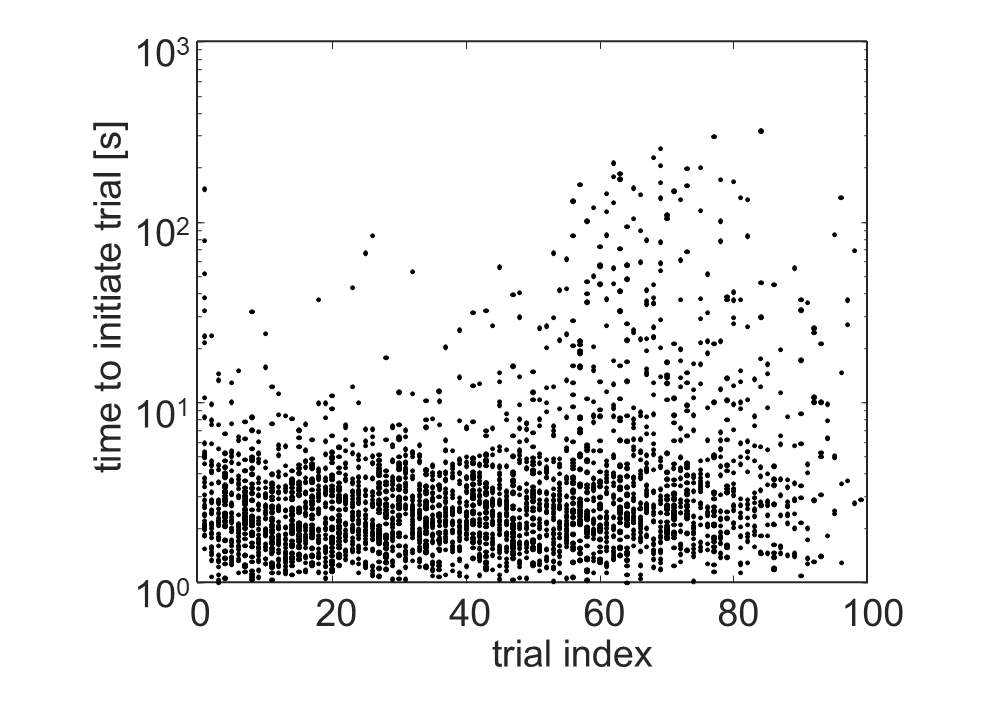

Supplement: SUPPLEMENTARY FIGURE S5 — Time to initiate trial. Time to initiate the trial for each trial from sessions in which this information was available (all sessions for CB5 and CB6, session #1-5 for CB10) from all three mice are superimposed. Note that each session has a different number of trials. Trials in which the time to initiate is shorter than 1 s are rare and omitted from this plot. [file Image_5.tif]
