# Supplementary material for: A novel behavioral paradigm using mice to study predictive postural control
Source: Front Neurosci. 2026 Apr 13;20:1790603. doi: 10.3389/fnins.2026.1790603 (PMC13111203; doi:10.3389/fnins.2026.1790603)
Supplement: Supplementary file 6 [file Table_1.docx]

**Table S1: Linear Mixed-Effects Model Coefficients and Statistics for Additional Mice (CB6 and CB10)**

**Mouse CB6**

|  | **Coefficients** | **Standard Errors** | **P-Values** |
| --- | --- | --- | --- |
| $\beta_{0}$ | 47.3 | 10.2 | $< 0.0001$* |
| $\beta_{cue}$ | −19.1 | 3.02 | $< 0.0001$* |
| $\beta_{trial}$ | 0.0049 | 0.047 | $0.92$ |
| $\beta_{session}$ | −0.38 | 0.84 | $0.65$ |

**Mouse CB10**

|  | **Coefficients** | **Standard Errors** | **P-Values** |
| --- | --- | --- | --- |
| $\beta_{0}$ | 78.4 | 8.43 | $< 0.0001$* |
| $\beta_{cue}$ | −11.7 | 3.28 | $0.00039$* |
| $\beta_{trial}$ | −0.086 | 0.039 | $0.026$* |
| $\beta_{session}$ | −3.15 | 0.79 | $< 0.0001$* |

* indicates significant value (p < 0.05)

**Table S2: Logistic Mixed-Effects Model Coefficients and Statistics for Additional Mice (CB6 and CB10)**

**Mouse CB6**

|  | **Exponentiated Coefficients** | **95% CI** | **P-Values** |
| --- | --- | --- | --- |
| $\beta_{0}$ | 0.83 | 0.19–3.56 | $0.80$ |
| $\beta_{1}$  (cue) | 3.04 | 1.93–4.80 | $< 0.0001$* |
| $\beta_{2}$  (trial) | 1.00 | 0.99–1.01 | $0.95$ |
| $\beta_{3}$  (session) | 1.02 | 0.90–1.15 | $0.77$ |

**Mouse CB10**

|  | **Exponentiated Coefficients** | **95% CI** | **P-Values** |
| --- | --- | --- | --- |
| $\beta_{0}$ | 0.15 | 0.04–0.58 | $0.0064$* |
| $\beta_{1}$  (cue) | 1.83 | 1.20–2.80 | $0.0051$* |
| $\beta_{2}$  (trial) | 1.01 | 1.00–1.01 | $0.0088$* |
| $\beta_{3}$  (session) | 1.17 | 1.02–1.33 | $0.021$* |

* indicates significant value (p < 0.05)
